# Editorial: Membrane domains as new drug targets

**DOI:** 10.3389/fphys.2015.00172

**Published:** 2015-06-02

**Authors:** Annemiek B. van Spriel, Geert van den Bogaart, Alessandra Cambi

**Affiliations:** Department of Tumor Immunology, Radboud Institute for Molecular Life Sciences, Radboud University Medical CenterNijmegen, Netherlands

**Keywords:** plasma membrane, microdomains, membrane lipids, lipid rafts, tetraspanin enriched microdomain, membrane receptors

Our understanding of the plasma membrane of eukaryotic cells has profoundly changed over the past 40 years (Kusumi et al., 2012). It is now well-understood that at the plasma membrane lipids and proteins are not randomly distributed, but are highly organized in discrete membrane domains with specific compositions such as lipid rafts, tetraspanin-enriched microdomains, caveolae or cortical actin-dependent membrane compartments (Cambi and Lidke, 2012; Kusumi et al., 2012). Membrane domains act as dedicated platforms for a whole range of cellular functions, such as cell proliferation, adhesion, migration, and intracellular trafficking, thus being appealing targets for drug treatment. This possibility has sprouted in numerous research efforts to identify and characterize drugs that selectively target membrane domains. Drugs may block or promote cellular functions by inducing, stabilizing, or disrupting the integrity or structure of these membrane domains.

This Review Topic provides a timely overview of the latest insights in the pharmaceutical potential of membrane domains, both protein- and lipid-based. Beckmann and colleagues highlight current research studies and clinical trials revolving around the inhibition of ceramide domain formation by antidepressants targeting the enzyme acid sphingomyelinase (ASM) (Beckmann et al., 2014). Considering the involvement of ASM-depending ceramide domains in several diseases including cancer (Petersen et al., 2013), infectious diseases (Grassme et al., 2003) and major depression (Gulbins et al., 2013), the impact of drugs that inhibit ASM activity is potentially broad (Kornhuber et al., 2010).

Milovanovic and Jahn review the roles of microdomains in neurobiology focusing on the molecular mechanisms mediating domain organization at the presynaptic membrane (Milovanovic and Jahn, 2015). The organization of SNARE proteins in membrane domains for localized vesicle docking, release and retrieval is a highly studied topic. SNARE proteins catalyze the final fusion steps of the neurotransmitter containing synaptic vesicles with the plasma membrane (Jahn and Scheller, 2006; Hong and Lev, 2014) and are the main targets of the Clostridial neurotoxins, which are widely used in the clinic as muscle relaxants (Rossetto et al., 2014). The authors not only provide an overview of the mechanisms that contribute to the clustering of SNARE proteins (van den Bogaart et al., 2013), but also integrate these into a single and elegant model describing the organization of SNAREs in membrane domains at the presynaptic membrane.

Navarro and co-workers discuss the therapeutic potential of targeting caveolae (Shvets et al., 2014) for treatment of cardiovasculature diseases, including atherosclerosis and pulmonary arterial hypertension (Navarro et al., 2014). Caveolae function in transcytosis of cholesterol-rich lipoproteins across endothelium of blood vessels and in the regulation of the activities of various ion channels and G-protein coupled receptors. Navarro and colleagues also review the connection between caveolae and Parkinson and Alzheimer diseases, where caveola are affected, and discuss how this may relate to the etiology and pathophysiology of these neurodegenerative diseases. The authors discuss potential strategies for developing drugs that selectively target caveolae.

Tetraspanins belong to the superfamily of four-transmembrane proteins that interact *in cis* with other membrane proteins and intracellular proteins forming so-called “tetraspanin microdomains” (Tarrant et al., 2003). These have attracted much attention as potential drug targets since recent studies have demonstrated that tetraspanins not only organize proteins in the plasma membrane, but also that they can directly regulate signaling pathways (Levy and Shoham, 2005; Lapalombella et al., 2012; van Spriel et al., 2012). Tetraspanin expression by tumor cells has been related to tumor progression and metastasis (Wright et al., 2004; Zoller, 2009; Hemler, 2014).

Ailane et al. describe the potency of targeting the tetraspanin Co-029/Tspan8 on colorectal tumor cells (Ailane et al., 2014). The expression profile of Tspan8 is largely restricted to squamous epithelial cells in the digestive tract, capillary endothelial cells, nerves, and muscle cells. Tspan8 over-expression has been documented in different carcinomas which relates to an increased metastatic capacity of tumor cells and poor prognosis (reviewed in Zoller, 2009). Here, the effect of a Tspan8 antibody on colorectal cancer cells was investigated in tumor cell lines and in a xenogeneic mouse model. Although no effect on tumor cell proliferation or apoptosis induction was found *in vitro*, antibody treatment of tumor-bearing mice resulted in significantly delayed tumor growth. Binding of the Tspan8 antibody to the tumor cells may modify their response to the tumor microenvironment possibly by disrupting the assembly of tetraspanin microdomains containing integrins, growth factor receptors, and/or signaling molecules.

The effectiveness of tetraspanin targeting in hematological malignancies is concisely reviewed by the group of Muthusamy (Beckwith et al., 2015). CD37 is highly expressed by B cells, B cell lymphomas and some leukemias. The historical view of tetraspanins as membrane organizers or scaffolds may be incomplete as it has recently been shown that CD37 has direct signaling capacities (Lapalombella et al., 2012). The intracellular tails of CD37 contain ITAM-like and ITIM-like motifs that may recruit PI3K and SHP-1. Coupling of CD37 to the Akt survival pathway has been shown in CD37-deficient mice that have defects in humoral immunity (van Spriel et al., 2012). Excitingly, several CD37-targeting therapeutics have been developed which are currently under evaluation in clinical trials in patients with B cell malignancies (Zhao et al., 2007; Heider et al., 2011; Deckert et al., 2013).

The use of transmembrane proteins as target for intracellular drug delivery is an exciting possibility discussed by Popov-Čeleketić and Van Bergen en Henegouwen, who elaborate on the role of cancer-specific growth factor receptor domains in receptor-mediated endocytosis (Popov-Celeketic and van Bergen En Henegouwen, 2014). Focusing on the epidermal growth factor receptor, the authors discuss how membrane receptor clustering and internalization can be exploited to target cancer cells using classical antibodies (Okines et al., 2011) and nanobodies (Heukers et al., 2014) conjugated to cytotoxic pharmaceutical agents.

Together, we believe our Review Topic provides an exciting overview of the various targeting and effector strategies of drugs that selectively target membrane domains. This synergistic overview brings together previously detached research fields and disciplines. Given the increasing appreciation of the fundamental importance of membrane domains in cellular function, we hope that this overview will foster the development or optimization of new pharmaceutical strategies for a wide range of human diseases and disorders.

## Conflict of interest statement

The authors declare that the research was conducted in the absence of any commercial or financial relationships that could be construed as a potential conflict of interest.
